# Complete Sequence and Molecular Epidemiology of IncK Epidemic Plasmid Encoding *bla*_CTX-M-14_

**DOI:** 10.3201/eid1704.101009

**Published:** 2011-04

**Authors:** Jennifer L. Cottell, Mark A. Webber, Nick G. Coldham, Dafydd L. Taylor, Anna M. Cerdeño-Tárraga, Heidi Hauser, Nicholas R. Thomson, Martin J. Woodward, Laura J.V. Piddock

**Affiliations:** Author affiliations: The University of Birmingham, Birmingham, UK (J.L. Cottell, M.A. Webber, D.L. Taylor, L.J.V. Piddock);; Veterinary Laboratories Agency, New Haw, Surrey, UK (N.G. Coldham, M.J. Woodward);; European Nucleotide Archive–European Bioinformatics Institute, Hinxton, UK (A.M. Cerdeño-Tárraga);; The Wellcome Trust Sanger Institute, Hinxton (H. Hauser, N.R. Thomson)

**Keywords:** Bacteria, Escherichia coli, antimicrobial drug resistance, extended-spectrum beta-lactamase, CTX-M, plasmid, epidemiology, research

## Abstract

Antimicrobial drug resistance is a global challenge for the 21st century with the emergence of resistant bacterial strains worldwide. Transferable resistance to β-lactam antimicrobial drugs, mediated by production of extended-spectrum β-lactamases (ESBLs), is of particular concern. In 2004, an ESBL-carrying IncK plasmid (pCT) was isolated from cattle in the United Kingdom. The sequence was a 93,629-bp plasmid encoding a single antimicrobial drug resistance gene, *bla*_CTX-M-14_. From this information, PCRs identifying novel features of pCT were designed and applied to isolates from several countries, showing that the plasmid has disseminated worldwide in bacteria from humans and animals. Complete DNA sequences can be used as a platform to develop rapid epidemiologic tools to identify and trace the spread of plasmids in clinically relevant pathogens, thus facilitating a better understanding of their distribution and ability to transfer between bacteria of humans and animals.

## Abstract 2

This plasmid is disseminated worldwide in *Escherichia coli* isolated from humans and animals.

Bacterial plasmids are key vectors of horizontal gene transfer, mediating the mobilization of genetic material from 1 bacterium to another. Their ability to capture DNA and to spread within and between bacterial species by conjugation facilitates the rapid dissemination of potentially beneficial genes through a bacterial population. These genes might alter virulence of the host, confer metabolic benefits, or enable the bacteria to colonize new environments (*1*). Genes that confer resistance to antimicrobial drugs used in human or veterinary medicine are often mobilized on plasmids. One class of resistance genes encode extended-spectrum β-lactamases (ESBLs), which confer resistance against many β-lactam antimicrobial drugs, leading to treatment failures (*2*). Within the past decade, cefotaximase-modifying (CTX-M) β-lactamases (CTX-M) have become the most prevalent ESBLs in bacteria isolated throughout the world in hospital and community settings (*3*). More than 85 variants have been identified (www.lahey.org/Studies), mainly in isolates of *Escherichia coli* that cause community-acquired urinary tract infections (*4*). Although clonal expansion events appear to have contributed to the spread of particular CTX-M variants, such as *bla*_CTX-M-15_ within *E. coli* strain O25:H4**-**ST131:05 (*5**,**6*), plasmids with the ability to spread efficiently, or epidemic plasmids, also are believed to be responsible for disseminating CTX-M ESBLs (*7*). The ability and frequency with which antimicrobial resistance genes disseminate between bacteria in humans, the environment, and animals is still debated, and the role of plasmids in this movement between ecosystems, including the food chain, is also still contested, despite mounting evidence that it occurs (*8**,**9*).

CTX-M-14 is the second most frequently identified CTX-M enzyme worldwide (*10*), detected in bacteria isolated from humans, animals, and the environment. CTX-M-14–producing strains show a high level of clonal diversity (*11**,**12*); therefore, dissemination has been attributed to conjugative plasmids rather than to clonal expansion of a bacterial host strain (*13*). In Europe, an association has been suggested between *bla*_CTX-M-14_ and plasmids of the incompatibility group IncK, or the spread of 1 particular IncK plasmid (*11**,**13**,**14*). In the United Kingdom in 2006, Liebana et al. described an ESBL-producing isolate from calves with diarrhea that carried *bla*_CTX-M-14_ on an IncK plasmid, denoted pCT (*15**,**16*). The plasmid spread to unrelated *E. coli* isolates within an index cattle farm and persisted within the environment. In this study, we report the full sequence and analysis of pCT and demonstrate the spread of pCT-like plasmids in animal and human *E. coli* isolates from the United Kingdom, Europe, Australia, and Asia.

## Materials and Methods

### Bacterial Strains

*E. coli* C159/11 was isolated from calves on a dairy farm in the United Kingdom in 2004 (*15**,**16*). Investigation and manipulation of the C159/11 plasmid (pCT) was conducted in an *E. coli* DH5α transformant created for the current study. We also investigated 15 CTX-M-14–producing *E. coli* isolates collected during 2006–2009 from cattle feces on farms in different geographic locations in the United Kingdom and 15 *E. coli* clinical isolates from England (P. Hawley, unpub. data), Germany (*17*), Spain (*11**,**18*), the People’s Republic of China (*19*), and Australia (*20*) (Table 1).

**Table 1:** CTX-M-14–producing *Escherichia coli* isolates used in this study*

Origin	Year	Location	Strain/plasmid	Inc type	Source
Cattle	2004	England/Wales	C159/11/ pCT	K	(15,16)
Cattle	2006	England/Wales	I779	F, K	NRL
Cattle	2008	England/Wales	I780	F, K	NRL
Cattle	2008	England/Wales	I781	FIA	NRL
Cattle	2009	England/Wales	I782	F	NRL
Cattle	2007	England/Wales	I783	Unknown	NRL
Cattle	2008	England/Wales	I784	Unknown	NRL
Cattle	2008	England/Wales	I785	Unknown	NRL
Cattle	2006	England/Wales	I786	I1-γ	NRL
Cattle	2006	England/Wales	I787	Unknown	NRL
Cattle	2008	England/Wales	I788	Unknown	NRL
Cattle	2008	England/Wales	I789	Unknown	NRL
Cattle	2006	England/Wales	I790	Unknown	NRL
Cattle	2008	England/Wales	I791	F	NRL
Cattle	2008	England/Wales	I792	F	NRL
Cattle	2008	England/Wales	I793	F	NRL
Human	No data	England	L125	Unknown	P. Hawkey, unpub. data
Human	2006	Germany	386	FII	(17)
Human	2006	Germany	400	FII	(17)
Human	2003–4	Spain	C574	K	(18)
Human	2003–4	Spain	C559	K	(18)
Human	2003–4	Spain	C567	K	(18)
Human	2001–5	Spain	FEC383/ pRYC105	K	(11)
Human	2002	Spain	E36/ pRYC110	HI2	(11)
Human	1998	People’s Republic of China	CH13/ pOZ174	Unknown	(19)
Human	2005–7	Australia	JIE 052	B	(20)
Human	2005–7	Australia	JIE 081	FII	(20)
Human	2005–7	Australia	JIE 084	FII	(20)
Human	2005–7	Australia	JIE 088	I1	(20)
Human	2005–7	Australia	JIE 182	B	(20)
Human	2005–7	Australia	JIE 201	K	(20)

*CTX-M, cefotaximase-modifying; NRL, National Reference Laboratory for Enteric Zoonotic Bacteria of Animal Origin of the Veterinary Laboratories Agency, New Haw, UK.

### Plasmid Extraction and Manipulation

Plasmid pCT was extracted from *E. coli* DH5α transconjugants by using an alkaline sodium dodecyl sulfate Maxi preparation (*21*) and cesium chloride density gradient centrifugation (*22*). Conjugation was by solid mating on a filter (Whatman, Maidstone, UK), by using rifampin-resistant *E. coli* (DH5α) as a recipient and selection of transconjugants on Luria-Bertani agar containing 50 µg/mL rifampin and 8 µg/mL cefotaxime.

### Antimicrobial Drug Susceptibility Testing

Susceptibility of C159/11 and pCT transconjugants to a panel of antimicrobial drugs (ampicillin, cefotaxime, cefoxitin, chloramphenicol, ciprofloxacin, naladixic acid, streptomycin, and tetracycline) was determined by using a microtiter broth double dilution method (www.bsac.org.uk/_db/_documents/Chapter_2_Determination_of_MICs_2006.pdf). Susceptibility of *E. coli* DH5α to the antimicrobial drugs tested was also determined.

### Complete Mucleotide Sequencing of pCT

The plasmid DNA sequence was determined by using a 454/Roche GS FLX analyzer (Roche, Basel, Switzerland). The de novo assembly generated 93631 bases in 7 contigs by using the 454/Roche Newbler assembly program with an N50 of 52,495 bp. The sequence represents improved high-quality draft sequence (*23*) with no discernable misassembles having undergone multiple rounds of computational gap closure. Annotation was completed by using Artemis (Sanger, Cambridge, UK). Further comparative analysis of the DNA sequence used Double ACT and Artemis Comparison Tool (Sanger, Cambridge, UK), DNAstar (Lasergene; Madison, WI, USA) and BLAST (www.ncbi.nlm.nih.gov/guide).

### PCR Amplification and Sequencing

Boiled bacterial cell lysates provided template DNA, 1 µL of which was added to PCR ReddyMix Master mixture (Abgene, Epson, UK). Typically, PCR conditions were 30 cycles of 95°C for 30 sec, 51°C for 30 sec, and 72°C for 30 sec. PCR was used to detect *bla*_CTX-M-14_ and insertion sequences ISE*cp1* and IS*903* as described (*24**–**26*). All primers used are shown in Table 2. To determine whether the pCT *bla*_CTX-M-14_ shares a common insertion site with *bla*_CTX-M-14_ on other plasmids, PCRs were designed to amplify the sequence from *bla*_CTX-M-14_ into both pCT flanking genes.

**Table 2:** Primers used for detecting pCT-like regions in plasmids from *Escherichia coli*, United Kingdom, Europe, Australia, and Asia, 2006–2009

Primer	Sequence, 5′ → 3′	Target DNA sequence	Size, bp	pCT binding site	Reference
CTX-M-G9 (F)	ATGGTGACAAAGAGAGTGCAAC	*bla*_CTX-M_ group 9 variants	876	70259–70280	(25)
CTX-M-G9 (R)	TTACAGCCCTTCGGCGATG	*bla*_CTX-M_ group 9 variants	876	69405–69423	(25)
ISEcp1A (F)	GCAGGTCTTTTTCTGCTCC	Insertion sequence ISEcp1	527	71728–71746	(27)
ISEcp1B (R)	ATTTCCGGAGCACCGTTTGC	Insertion sequence ISEcp1	527/1,037†	71220–71239	(27)
B3A (F)	AACGGCACAATGACGCTGGC	Insertion sequence IS903	887	69913–69932	(24)
IS903 (R)	TGTAATCCGGCAGCGTA	Insertion sequence IS903	887	69045–69061	(24)
Pseudo (R)	AACATTCGGCCGTTCACAGC	Region downstream of *bla*_CTX-M-14_	1,636	68644–68663	This study
*traK* (F)	GGTACCGGCATCGCACAGAA	Region upstream of ISEcp1	1,037	72238–72257	This study
Sigma (F)	ACAGCGTCTTCTCGTATCCA	pCT putative sigma factor	1,289	48590–48609	This study
Sigma (R)	GTTCTTCCAGCTGACGTAAC	pCT putative sigma factor	1,289	47320–47339	This study
pCT *rci* (F)	AAGGTCATCTGCAGGAGT	pCT shufflon recombinase	945	78364–78381	This study
pCT *rci* (R)	GTGTGCGCAGCAACAATA	pCT shufflon recombinase	945	77436–77453	This study
*pilN* (F)	GACAGGCAGAGAACACCAGA	pCT *pilN* outer membrane protein	627	88267–88286	This study
*pilN* (R)	ATGCTGTTCCACCTGATGAG	pCT *pilN* outer membrane protein	627	87659–87678	This study
*nikB* (F)	CGTGCMTGCCGTGARCTT	IncI complex *nikB* relaxase gene	290	33077–33094	This study
*nikB* (R)	TCCCAGCCATCCWTCACC	IncI complex *nikB* relaxase gene	290	33350–33367	This study
pCT008 (F)	CATTGTATCTATCTTGTGGG	pCT pCT008-pCT009 region	428	3665–3684	This study
pCT009 (R)	GCATTCCAGAAGATGACGTT	pCT pCT008-pCT009 region	428	4074–4093	This study

*pCT, IncK plasmid; CTX-M, cefotaximase-modifying; F, forward primer; R, reverse primer. †Primer ISEcp1B can be paired with primer ISEcp1A (527 bp) or with primer traK (1,037 bp).

Using the pCT sequence, we designed primer pairs to amplify novel regions of pCT for rapid identification of potential pCT-like plasmids in CTX-M-14–producing bacteria (Table 2). PCRs to amplify DNA encoding the putative sigma factor, *pilN* gene, and shufflon recombinase were developed into a multiplex PCR. An additional primer pair was designed to a unique region of pCT and compared with other known sequences for amplification across coding sequences (CDSs) pCT008–pCT009 for further discrimination of pCT-like plasmids. Sequencing of the relaxase gene has been reported to further categorize plasmids within the IncI complex (*11**,**28*). A modified primer pair was designed for this region (*nikB*) by using sequence data from pCT and other related sequenced plasmids. Resulting amplicons were sequenced by using BigDye Terminator version 3.1 cycle sequencing (Applied Biosystems, Foster City, CA, USA) at the functional genomics laboratory of the University of Birmingham (Birmingham, UK) sequences were aligned by using MEGA 4.0 (*29*) for phylogenetic analysis (*30*). Primers amplifying group 9 *bla*_CTX-M_ genes were used as a positive control in each instance. The complete DNA sequence of plasmid pCT was assigned GenBank accession nos. F868832 and NC_014477.1.

## Results

### Features of pCT

**Figure 1:**
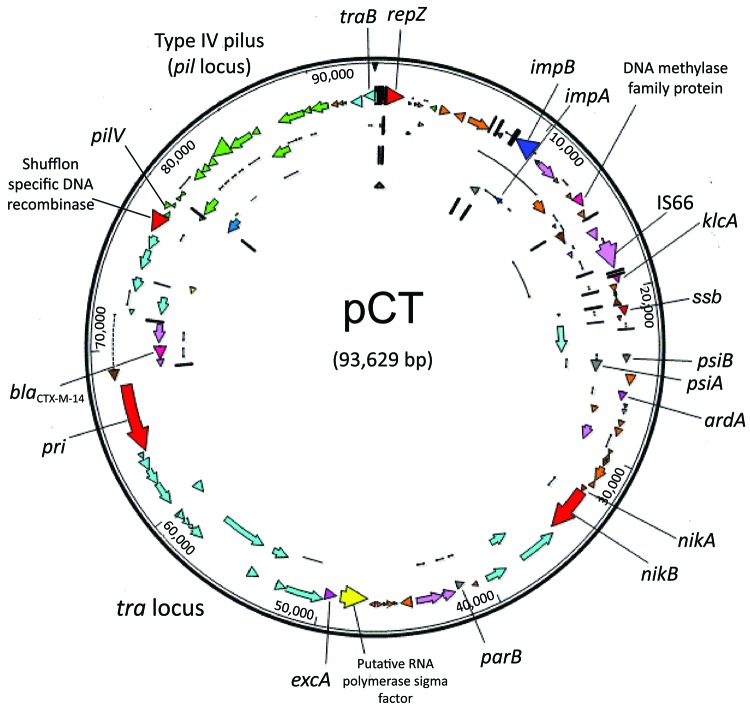
Circular map of plasmid pCT. Open reading frames are color coded as follows: brown, pseudogenes; orange, hypothetic proteins; light pink, insertion sequences; light blue, *tra* locus; green, *pil* locus; dark pink, antimicrobial drug resistance gene; yellow, putative sigma factor; red, replication-associated genes. Arrows show the direction of transcription. pCT, IncK plasmid.

The *bla*_CTX-M-14_–carrying plasmid isolated from *E. coli* C159/11 was demonstrated to be conjugative by successful transfer to *E. coli* DH5α by using filter mating and previously determined to be of the incompatibility group IncK (*16*). Analysis of transconjugants showed resistance to β-lactam antimicrobial drugs as the only transferrable resistance phenotype. Whole-plasmid sequencing showed that pCT was 93,629 bp (Figure 1) with an average G+C content of 52.67%. Annotation of the plasmid showed 115 potential protein CDSs, 89 of which were homologous to proteins of known function (Technical Appendix ). No genes known to play a role in determining virulence were identified, and sequencing confirmed *bla*_CTX-M-14_ as the only antimicrobial drug resistance gene on pCT. The pCT *bla*_CTX-M-14_ gene is found between insertion sequences ISE*cp1* and IS*903* as described (*31*).

Most of the identified putative coding open reading frames are typical for an IncI group plasmid backbone (*32*). Two conjugal transfer systems were identified. The first is the more commonly described *tra* operon, which encodes the primary pilus for conjugation transfer. The pCT *tra* locus is analogous to the *tra* operon of R64/ColIb-P9 and is found in a similar conformation, although minor differences existed between the 3 plasmids throughout. The second is the *pil* locus encoding a thin pilus, which is believed to increase conjugation rates in liquid media. The tip of this thin pilus is variable, and the exact nature of the expressed epitope is determined by the orientation and order of *pilV* shufflon components (pCT_094-pCT_098), which can be inverted by the action of the recombinase protein (pCT_093) encoded downstream. Analysis of the pCT plasmid assembly showed that this region was present in multiple forms (data not shown), which is consistent with site-specific recombination mediated by a shufflon recombinase. The pCT shufflon potentially differs from that of the other closely related plasmids (pO113, pO26_vir and pSERB1) because each of these apparently has an inactive shufflon, which can be attributed to the absence of the recombinase or an insertion element within this CDS. The antirestriction and segregation genes on pCT are typical of this type of plasmid.

### Comparison of pCT with Other Plasmids

**Figure 2:**
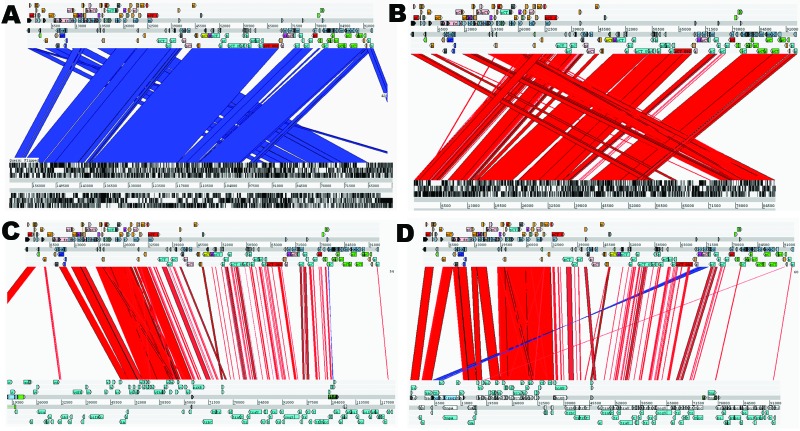
Artemis Comparison Tool (Sanger, Cambridge, UK) comparisons of IncK plasmid (pCT) with other plasmids. Complete DNA sequence plasmid comparisons. Bands of color indicate homology between sequences. Red lines show sequence in the same confirmation; blue lines indicate sequence inversion. The pCT sequence is represented as the top line of each comparison compared with pO26_vir (GenBank accession no. FJ38659) (A); R387 (B); R64 (accession no. AP005147) (C); and pEK204 (accession no. EU935740) (D) on each bottom line.

When we compared complete sequences deposited in GenBank of plasmids carrying *bla*_CTX-M_ genes with pCT, we found that outside the *bla*_CTX-M_ gene those plasmids with different replication mechanisms, such as those within the IncFII group or of the IncN group (pKP96) (*33*), have almost no DNA homology to pCT. The only other *bla*_CTX-M_ carrying IncI complex plasmid to be sequenced and deposited in GenBank thus far is a *bla*_CTX-M-3_ carrying IncI plasmid pEK204 (EU935740) (*32*). pEK204 shares sequence conservation over ≈60% of the pCT genome, including most of the core IncI complex–related genes for replication or transfer. Further similarities were found in the minimal carriage of resistance genes (Figure 2, panel D).

The pCT genome was also compared with other IncI group sequenced plasmids to identify regions considered core or backbone and to determine novel encoded genes. IncI complex reference plasmids R64 (AP005147) and ColIb-P9 (AB021078) shared 99% identity with 64% of the pCT sequence primarily within genes involved in replication and conjugation (Figure 2, panel C). Other plasmids compared with pCT included R387, the Sanger IncK reference plasmid, which shared a high percentage identity to pCT across IncK-specific core genes (Figure 2, panel B).

We investigated novel genes by identifying regions of the pCT sequence absent from those plasmid sequences with most homology to pCT: pO26_vir (FJ38659), pO113 (AY258503), and the partially sequenced pSERB1 (AY686591), none of which carry *bla*_CTX-M_ genes. Both pO26_vir (168,100 bp) and pO113 (165,548 bp) are large plasmids that carry several virulence genes and share 99% homology with 85% and 83% of the pCT genome, respectively (Technical Appendix Table; Figure 2, panel A). pSERB1 also has high DNA sequence identity (91% of the pSERB1 deposited genome sequence has 99% identity to two thirds of the pCT sequence), however, because this plasmid is deposited in GenBank only as a partial sequence, the total identity cannot be assessed.

### Detection of pCT-like Plasmids in Animal and Human Isolates

**Figure 3:**
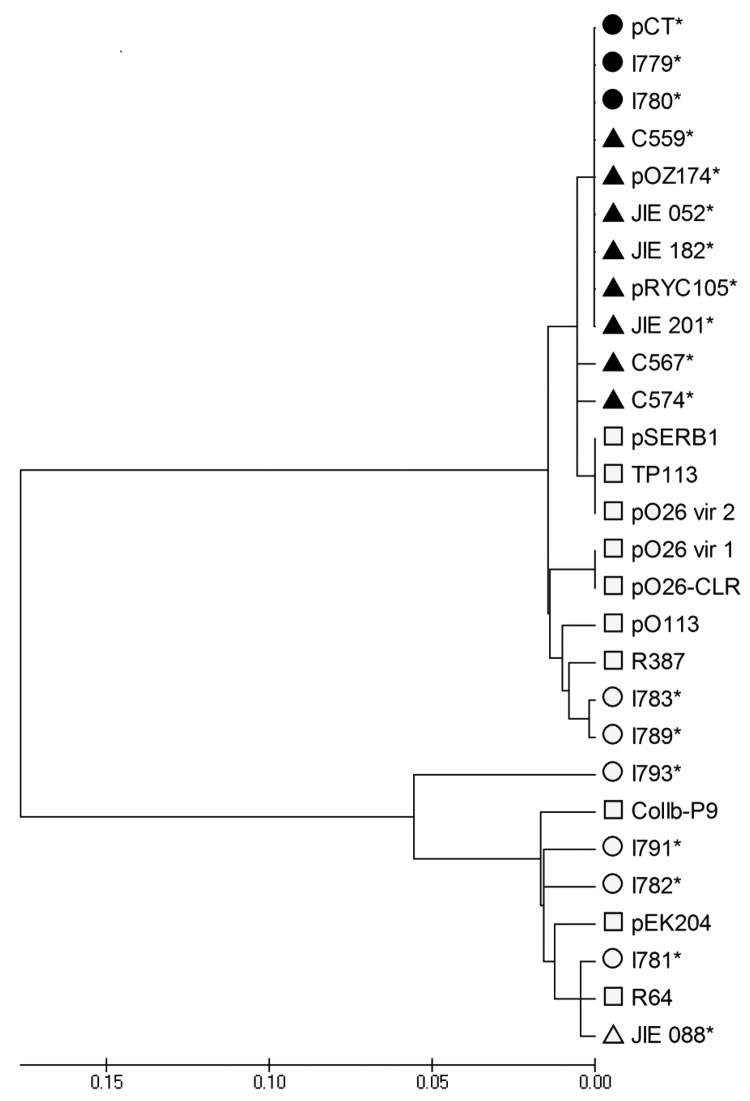
Phylogenetic analysis of *nikB* in IncI complex plasmids from *Escherichia coli.* DNA sequences of *nikB* PCR amplicons and sequences obtained from public resources were aligned and analyzed by using MEGA 4.0 (*29*). A neighbor-joining tree was constructed by using complete deletion modeling and computed by using the maximum composite likelihood method (*30*). The phylogenetic tree was linearized assuming equal evolutionary rates in all lineages. Circles, *nikB* sequences from plasmids isolated from veterinary isolates from the United Kingdom; triangles, *nikB* sequences of plasmids from *Escherichia coli* isolated from humans; squares, *nikB* sequences of plasmids obtained from GenBank or the Sanger Institute; shaded shapes, plasmids identified as pCT-like by using PCR in this study; asterisks, plasmids encoding *bla*_CTX-M-14_. pCT, IncK plasmid. Scale bar indicates nucleotide substitutions per site.

Fifteen CTX-M-14–producing *E. coli* isolates collected from different cattle farms around the United Kingdom were examined for pCT-like plasmids with a series of PCRs that amplified characteristic regions of pCT. Veterinary isolates I779 and I780 were obtained 2 years apart and from different geographic areas in the United Kingdom. These isolates carried plasmids encoding all 6 pCT regions that were investigated (*bla*_CTX-M-14_, *nikB*, putative sigma factor, *rci*, *pilN*, and pCT008–009) and when compared with pCT had identical flanking regions adjacent to *bla*_CTX-M-14_. Therefore, these plasmids were deemed pCT-like. Fifteen CTX-M-14–producing *E. coli* human clinical isolates from England, Germany (*17*), Spain (*11**,**18*), Australia (*20*), and China (*19*) also were examined for pCT (Table 1). No pCT-like plasmids were detected in the isolates from the United Kingdom and Germany. However, pCT-like plasmids were identified in 4 of 5 clinical isolates from Spain (C559; C567; C574; FEC383), 3 of 6 isolates from Australia (JIE 052, JIE 182, JIE 201), and the isolate from China (*E. coli* 8, CH13) because all sequences specific to pCT could be amplified by PCR. pCT-like plasmids have also been shown to be present in other clinical *E. coli* isolates from the United Kingdom by using the same multiplex assay (M. Stokes et al., pers. comm.). Sequencing of amplicons generated during PCR amplification of *nikB* showed pCT-like plasmids had *nikB* sequences with >98% DNA identity to the pCT *nikB* sequence and clustered when these sequences were used to construct a phylogenetic tree (Figure 3). *nikB* sequences from non–pCT-like plasmids clustered further from pCT within the phylogenetic tree (Figure 3). This analysis further supports the hypothesis that pCT has disseminated broadly between bacteria in animal and human ecosystems.

## Discussion

We report the complete sequence of a *bla*_CTX-M-14_–carrying IncK plasmid, pCT, from an *E. coli* isolate from a cattle farm in the United Kingdom (*16*). Within the 115 putative CDs, there is an absence of any genes known to play a role in determining virulence of the host and the absence of any other antimicrobial drug resistance genes except for *bla*_CTX-M-14_. Therefore, the persistence and spread of pCT cannot be attributed to coselection associated with pressure from non–β-lactam antimicrobial drugs. This finding suggests that pCT persistence and dissemination have been driven by either constant β-lactam exposure or that pCT can remain stable within a population in the absence of any antimicrobial drug selective pressure. There has been much speculation about the role of the type IV pilus and its shufflon found in plasmids and possible role(s) in adhesion of the host bacterium to surfaces and eukaryotic epithelial cells in vitro and in biofilm formation (*34*). The thin pilus might also aid conjugation in a liquid environment by anchoring donor and recipient cells (*35*). This system may have played a role in the persistence of *E. coli* C159/11, which originally was found to remain within slurry and on the floor of cow sheds during the longitudinal farm study from which it was identified (*16*). These attributes of the type IV pilus might contribute to the persistence of pCT within bacteria isolated from the UK farm and in animals and humans throughout the world.

Two other features of pCT are of interest. The first, and most notable, is a putative RNA polymerase sigma factor (CDS pCT_066) within the sequence, which shares homology with genes found in only 4 closely related plasmids (pO26vir; pO113, TP113, and pSERB1) and has limited identity to homologue SigB in *Yersinia frederiksenii.* Other weak protein matches show some homology to the extracytoplasmic function sigma factors, small regulatory proteins divergent in sequence to most of the other sigma factors and involved in global gene regulation. Both examples are chromosomally encoded. Although sigma factors of this group have previously been noted on plasmids, scant information has been published about their role or function.

The second feature of interest is that in large stably maintained conjugative plasmids, such as R64, functional large addiction operons such as ParA/B or *kor/mck* usually are present; however, these are lacking in pCT. Despite the apparent lack of stability or persistence genes, pCT has remained stable in a population in the absence of selective pressure for prolonged periods (N.G. Coldham et al., unpub. data).

Comparison of the genome of pCT with other *bla*_CTX-M_–encoding plasmids showed no conserved regions outside the β-lactamase gene. Therefore, no single feature of the plasmid backbone appears responsible for the spread of *bla*_CTX-M_ genes, and the acquisition of these genes is unlikely to have been a single event. Homology was highest between pCT and 4 plasmids (pO26_vir, pO113, pSERB1, and TP113). pO26_vir was identified in a Shiga toxin–producing *E. coli* strain 026:HII and encodes several virulence genes not found on pCT, including genes for the production of a hydrolase, catalase, and a hemolysin transport protein. pO113 was isolated from another hemolysin-producing EHEC O113:H21 *E. coli* sample from a patient in Australia (*36*). The finding that pCT is most closely related to 2 plasmids that carry an array of virulence genes is of concern because of the potential for recombination between these plasmids, creating mobile elements carrying virulence genes and the *bla*_CTX-M-14_.

The genome sequencing of pCT enabled development of PCRs that amplified discrete regions of the pCT sequence, thereby enabling rapid identification of other pCT-like plasmids that share these loci. pCT-like plasmids were identified in bacteria isolated from 2 other UK farms in 2006 and 2008 and, most recently, from human clinical isolates in the United Kingdom (M. Stokes et al., pers. comm.).

Four human clinical isolates from Spain also carried pCT-like plasmids, with all 6 pCT regions amplified by PCR, which had the same insertion sites for *bla*_CTX-M-14_. These data show the ability of a large conjugative plasmid to transfer between bacteria isolated from humans and animals, facilitating the movement of *bla*_CTX-M-14_ between these niches. Since 2000, when CTX-M-14 was identified in bacteria from Spain, it has become one of the most commonly detected enzymes isolated from human and animal isolates in Spain (*24**,**37*). Previous studies conducted in hospitals in Spain examined an association between *bla*_CTX-M-14_ and IncK plasmids. Valverde et al. (*1**1*) isolated an IncK plasmid, pRYC105, from many lineages of *E. coli* from community-acquired infections and the environment in different geographic regions of Spain. These authors hypothesized that pRYC105 shared identity with the plasmid isolated in the United Kingdom by Liebana et al. (*16*), and the present study has confirmed this hypothesis by showing that pRYC105 is pCT-like.

Human clinical isolate *E. coli* 8 CH13, described in 2002 and isolated in 1998 from China, contained pOZ174, which encodes *bla*_CTX-M-14_ (*19*); as with pRYC105, we showed that pOZ174 is pCT like. Furthermore, our data suggest that pCT has persisted since 1998 and is distributed across Europe, Asia, and Australia in diverse *E. coli* lineages isolated from humans and animals. Because CTX-M-14 is the most frequently identified ESBL in Spain and China, further investigation using this molecular test will determine whether pCT is the dominant vector of *bla*_CTX-M-14_ in these areas and whether pCT has disseminated to other ecosystems. The identical insertion site for *bla*_CTX-M-14_ in each of the pCT-like plasmids investigated in our study suggests a single capture of this β-lactamase gene onto the plasmid backbone and subsequent spread of the plasmid.

The alignment and analysis of *nikB* from pCT-like plasmids were also used to determine how related the plasmids are and demonstrated sequence identity of >98%. These sequences clustered with pCT within a phylogenetic tree, which indicated less sequence divergence than with other IncI complex non–pCT-like plasmids. Design of the pCT–specific PCRs distributed throughout the plasmid and sequencing of *nikB* amplicons provided a useful and rapid tool in first identifying pCT-like plasmids. Relaxase or *nikB* typing also would provide a suitable locus in recently developed plasmid multilocus sequence typing. These assays can now be used to screen CTX-M-14–producing bacteria for other pCT-like plasmids.

The sequence of pCT enabled an understanding of its backbone and seems to suggest that, apart from plasmid replication and transfer functions, the only known gene that confers a selective advantage on this plasmid is *bla*_CTX-M-14_. Subsequent PCRs successfully indicated that pCT-like plasmids are distributed over several countries worldwide in bacteria isolated from humans and animals. This approach can be applied to the study of other plasmids of clinical relevance and facilitate better trace analyses of horizontally acquired antimicrobial drug resistance or virulence genes. Additionally, use of this method may lead to identification of new vectors and increase understanding of the interaction among bacteria isolated from humans, animals, and the environment.

## Supplementary Material

Technical Appendix Complete Sequence and Molecular Epidemiology of IncK Epidemic Plasmid Encoding blaCTX-M-14.
