# Complete Sequence and Molecular Epidemiology of IncK Epidemic Plasmid Encoding *bla*_CTX-M-14_

**DOI:** 10.3201/eid1707.C11707

**Published:** 2011-07

## Vol. 17, No. 4

References were misnumbered in the article Complete Sequence and Molecular Epidemiology of IncK Epidemic Plasmid Encoding *bla*_CTX-M-14_ (J.L. Cottell, et al.). The article has been corrected online (http://www.cdc.gov/eid/content/17/4/645.htm).

