# Supplementary material for: Complete Sequence and Molecular Epidemiology of IncK Epidemic Plasmid Encoding blaCTX-M-14
Source: Emerg Infect Dis. 2011 Apr;17(4):645–52. doi: 10.3201/eid1704.101009 (PMC3377399; doi:10.3201/eid1704.101009)
Supplement: Technical Appendix — Complete Sequence and Molecular Epidemiology of IncK Epidemic Plasmid Encoding blaCTX-M-14. [file 10-1009-Techapp.pdf]

Article DOI: 10.3201/eid1704.101009

Complete Sequence and Molecular Epidemiology of IncK Epidemic Plasmid Encoding *bla*$_{CTX-M-14}$

Technical Appendix

Technical Appendix Table. Protein coding regions of pCT listed clockwise around the plasmid map

Gene name	ID	Direction of coding	Coordinates	No. aa	DNA identity pO26-vir, %	DNA identity CoIIb-P9, %	Protein homolog	Function	Notes
repY	pCT_001	→	610–699	29	100	100	RepY	Positive regulator for *repZ*	
repZ	pCT_002	→	687–1718	343	100	100	RepZ	Replication initiator protein	
	pCT_003	→	2050–2199	49	100	95		Uncharacterised protein	
repA†	pCT_004	→	2193–2267<br>2267–2440	128	96	94	RepA4	Replication initiator protein	*repA4* found in R100
	pCT_005	→	2416–2574	51	90	9		Hypothetical protein	
yacA	pCT_006	→	2629–2898	89	94	97	YacA	Putative stabilisation protein	Putative homologues in IncPβ plasmid B8 of toxin-antitoxin system
yacB	pCT_007		2895–3176	93	96	97	YacB	Putative stabilisation protein	
	pCT_008	←	3372–3713	113	0	0		Putative membrane protein	
	pCT_009	→	4013–4600	195	0	0		Putative serine acetlyltransferase	
yafB	pCT_010	→	4882–5475	197	95	95	YafB	Metabolic proteins	
yagA	pCT_011	→	5829–7175	448	99	98	YagA	Replication and repair protein	
†	pCT_012	←	7710–8021	308	100	0		Hypothetical protein	
parA like	pCT_013	←	8009–8635	209	100	0	ParA	Putative stability protein	
impB	pCT_014	←	8847–10118	423	100	99	ImpB	UV protection protein	Similar arrangement to *imp* locus found in plasmid TP110
impA	pCT_015	←	10118–10555	145	100	99	ImpA	UV protection protein	
impC	pCT_016	←	10552–10800	82	100	99	ImpC	UV protection protein	
	pCT_017	→	10904–12124	1406	0	0		Transposase	
yccA	pCT_018	→	12236–12505	89	100	100	YccA	Inner membrane protein	
	pCT_019	→	12474–13445	323	95	97		Hypothetical protein	
	pCT_020	→	13449–13754	101	98	91		DNA methylase family protein	
	pCT_021	→	13830–14513	227	98	90		Hypothetical protein	
	pCT_022	→	14514–14735	73	99	90		Hypothetical protein	
†	pCT_023	→	14449–15183	144	95	92		Hypothetical protein	
ycfA†	pCT_024	→	15183–15197<br>15196–15228<br>15225–15863<br>15866–16000	273	100	0	YcfA	Conserved hypothetical plasmid protein	

IS66 A	pCT_025	→	16037–16714	225	0	0	IS66	Transposase	Also found in plasmid pEK204 disrupting the shufflon region
IS66 B	pCT_026	→	16714–17961	115	0	0	IS66	Transposase	
IS66 C	pCT_027	→	17081–18652	523	0	0	IS66	Transposase	
klcA	pCT_028	→	19125–19550	141	95	93	KlcA	Stable inheritance protein	
	pCT_029	→	19597–20019	140	98	96		Hypothetical protein	
†	pCT_030	←	20071–20214<br>20214–20369	100	95	94		Hypothetical protein	
	pCT_031	→	20410–20856	157	97	98		Hypothetical protein	
ssb†	pCT_032	→	20774–20938	143			Ssb	ssDNA binding	
ssb	pCT_033	→	20977–21504	175	99	95	Ssb	ssDNA binding	Gene involved in replication
	pCT_034	→	21761–21792	86	99	95	YkfF	conserved hypothetical plasmid protein	
ycjA	pCT_035	→	21761–23812	654	94	93	ParB	Partitioning protein	
psiB	pCT_036	→	23864–24301	145	99	97	PsiB	Plasmid SOS inhibition protein	Gene involved in replication
psiA	pCT_037	→	24298–25017	239	99	97	PsiA	Plasmid SOS inhibition protein	Gene involved in replication
	pCT_038	→	25014–25610	198	98	0		Hypothetical protein	
ardA	pCT_039	→	26972–26572	166	98	100	ArdA	Antirestriction protein	Gene involved in replication
†	pCT_040	→	26754–26969	155	100	97		Hypothetical protein	
	pCT_041	→	27093–27308	71	99	89		Hypothetical protein	
	pCT_042	→	27305–27739	144	99	98		Hypothetical protein	
ygdA	pCT_043	→	27736–28098	120	99	90	YhdA	Hypothetical protein	
ccgAII	pCT_044	→	28200–28664	129	100	0	CcgAII		
Transposase	pCT_045	→	28661–29551	296	98	89		Putative transposase	
ydgA	pCT_046	→	29551–29796	81	93	88		Putative	
ygeA	pCT_047	←	29827–30078	83	98	98		Putative	
ydhA†	pCT_048	→	30163–30183<br>30183–30533	123	99	97	YdhA		
ydiA	pCT_049	→	30658–31506	282	99	96	YdiA	Putative	
yggA	pCT_050	←	31593–31934	113	98	97	YggA	Putative	
nikA	pCT_051	→	32161–32493	110	100	71 (91)	NiKA	Relaxome component	
nikB	pCT_052	→	32505–35225	906	100	71 (91)	NikB	Relaxase	
trbC	pCT_053	←	35446–37746	766	99	76 (85)	TrbC	Putative prepillin	
trbB	pCT_054	←	37727–38851	374	100	69 (41)	TrbB	ATPase	
trbA	pCT_055	←	38848–40110	420	100	72 (86)	TrbA	Regulation	
	pCT_056	→	40742–41038	98	99	86		Hypothetical protein	
parB	pCT_057	←	41427–41894	155	99	0	ParB	Partitioning protein	No homology to other IncI *parB* genes
Transposase	pCT_058	←	42085–42840	251	0	0		Putative transposase	
Transposase	pCT_059	←	42857–44392	511	0	0		Putative transposase	
	pCT_060	→	44698–45321	207	99	0		Hypothetical protein	
	pCT_061	←	45519–45734	71	100	0		Hypothetical protein	
	pCT_062	←	45738–46106	122	99	0		Hypothetical protein	
	pCT_063	←	46118–46309	63	98	0		Hypothetical protein	
	pCT_064	←	46397–46717	106	98	0		Hypothetical protein	
pndC	pCT_065	→	46821–47114	97	100	91	PndA	Stabilisation protein	Putative post segregational killing gene

Sigma factor	pCT_066	←	47259–48845	528	99	0		Putative sigma factor	Protein homology to SigB, (Sigma 70 family) found in a *Yersinia frederiksenii* (ZP_04633307)
excA	pCT_067	←	49119–49772	215	100	68 (49)	excA	Surface exclusion protein	
traY	pCT_068	←	49860–52058	721	100	68	TraY	Integral membrane protein	
traX	pCT_069	←	52100–52669	189	100	72 (23)	TraX	Pilin	
traW	pCT_070	←	52666–53706	346	100	75	TraW	Transfer related lipoprotein	
traV	pCT_071	←	53829–54449	206	100	69	TraV		
traU	pCT_072	←	54449–57493	1,014	100	72	TraU	Nucleotide binding protein	
traT	pCT_073	←	57788–58501	237	100	68 (66)	TraT		
traS	pCT_074	←	58521–58772	83	100	0	TraS		
traR	pCT_075	←	58829–59227	132	100	0	TraR		
traQ	pCT_076	←	59274–59804	176	100	74	TraQ		
trap	pCT_077	←	59801–60514	237	100	74 (22)	TraP		
traO	pCT_078	←	60511–61848	445	100	71 (41)	TraO	Pore protein	
traN	pCT_079	←	61852–62826	324	100	75 (80)	TraN	Secretin/pore protein	
traM	pCT_080	←	62837–63532	231	100	73 (73)	TraM		
traL	pCT_081	←	63544–63894	116	100	72	TraL		
sogL	pCT_082	←	63911–67972	1,353	100	74 (54)	SogL/TraC	DNA primase	
†	pCT_083	←	68326–68922<br>72048–72083	232	0	0		Uncharacterised protein	39.53% G/C unlike surrounding CDS's
IS903B	pCT_084	←	68888–69370	322	0	0		Putative transposase	
$bla_{\text{CTX-M-14}}$	pCT_085	←	69405–70280	291	0	0	CTX-M-14	B-lactamase	$bla_{\text{CTX-M-14}}$ 249bp downstream (not usual 49bp)
ISEcp1	pCT_086	←	70530–71792	420	0	0		Transposase	
traK	pCT_087	←	72204–72494	96	100	69	TraK		
traJ	pCT_088	←	72491–73639	382	100	75	TraJ	Nucleotide binding protein	
traI	pCT_089	←	73623–74288	276	100	72 (81)	TraI	Transfer related lipoprotein	
traH	pCT_090	←	74326–74913	195	100	64 (66)	TraH		
traF	pCT_091	←	75017–76219	400	100	72	TraF		
traE	pCT_092	←	76321–77142	273	100	66 (72)	TraE		
Rci	pCT_093	←	77355–78479	474	97 (16)	72 (74)	Rci	Shufflon recombinase	
Shufflon A	pCT_094	→	78534–78893	119	99 (31)	66 (68)		PilV pilus protein A	Although detailed in IncI plasmid R64, there is only moderate resemblance of this system to the pCT shufflon. The entire region has most DNA homology (60%) to the shufflon region of plasmid R721.
Shufflon	pCT_095	←	78898–79287	129	98	0		PilV pilus protein C′ (alternative 3′ tip)	
Shufflon C'	pCT_096	→	79298–79528	76	0	83 (98)		PilV pilus protein C′ (alternative 3′ tip)	
Shufflon C	pCT_097	←	79525–79788	87	0	79 (97)		PilV pilus protein C′ (alternative 3′ tip)	
Shufflon B	pCT_098	→	79799–80062	87	0	70		PilV pilus protein C′ (alternative 3′ tip)	
pilV	pCT_099	←	80072–81337	421	99	73 (13)	PilV	Type IV prepilin	
pilU	pCT_101	←	81355–81981	208	99	0	PilU	Prepilin peptidase	
pilT	pCT_102	←	81997–82482	161	100	0	PilT	Transglycosylase	
pilS	pCT_103	←	82577–83063	178	100	0	PilS	Type IV prepilin	

pilR	pCT_104	←	83125–84219	364	99	0	PilR	Pilus biogenesis	
pilQ	pCT_105	←	84221–85729	502	99	74 (4)	PilQ	Nucleotide binding protein	
pilP	pCT_106	←	85832–86290	152	99	0	PilP	Pilus biogenesis protein	
pilO	pCT_107	←	86280–87575	431	99	0	PilO	Pilus biogenesis protein	
pilN	pCT_108	←	87596–89215	539	99	83 (2)	PilN	Secretin	
pilM	pCT_109	←	89247–89684	145	99	0	PilM	Pilus biogenesis protein	
pilL	pCT_110	←	89688–90758	356	99	0	PilL	Lipoprotein	
	pCT_111	←	90985–91341	122	100	0		Hypothetical protein	
pilI	pCT_112	←	91437–91679	80	100	0	PilI		
†	pCT_113	←	91760–91864	564	100	0		Hypothetical protein	
traC	pCT_114	←	92170–92832	220	94	0	TraC	Regulator of transfer	
traB	pCT_115	←	92972–93613	213	99	67 (81)	TraB	Regulator of transfer	

*pCT, IncK plasmid ; aa, amino acid; UV, ultraviolet; ss, single-stranded; SOS, DNA repair proteins; ATP, adenosine triphosphate.
†Pseudogene; DNA identify to pO26_vir and ColIb-P9 similarity (coverage).
